# Fatalism and malaria elimination: A historical perspective from Palestine 100 years ago

**Published:** 2018-05-01

**Authors:** Anton Alexander

**Affiliations:** 1 BC Business Centrum, Elscot House, Arcadia Avenue, London N3 2JU, UK

## Abstract

Fatalism is the acceptance of all things and events as inevitable. This sense of inevitability about malaria has obstructed malaria elimination from the outset and this article examines how this attitude was overcome in Palestine a century ago to enable the first start anywhere in the world of a successful malaria elimination campaign. The Balfour Declaration had been issued by the British Government in 1917 in support of a Jewish homeland in Palestine even though the British would have been aware Palestine was drenched in malaria and that Palestine was either uninhabitable in many areas or otherwise generally thinly populated. The only experience at that date of dealing with malaria control anywhere in the world had been demonstrated by General Gorgas at the Panama Canal together with his employment of thousands of men at vast expense, thus making it a method too costly to adopt for most countries. Notwithstanding this, Louis Brandeis, president of the American Zionists, had a strong commitment to grasp the moment provided by the Balfour Declaration, and to bring about a habitable Jewish homeland. Despite the pessimism and negativity of the rest of the Zionist establishment, which viewed malaria as a natural incident of Palestinian life, Brandeis prevailed upon Dr. Israel Kligler, a Zionist and also a brilliant public health scientist, to consider a fresh affordable method of controlling and eliminating malaria, and to thereby render Palestine habitable for Jewish settlement. Kligler’s significant change in approach against the disease was to think not of malaria control and use of thousands of employed personnel, but to seek instead malaria elimination through involvement of the community through culturally-sensitive education. Only absence of fatalism made this possible.

## 1 Introduction

For cynical readers who consider fatalism does not exist or is easily overcome, the following example may assist in relating to the problem. Fatalism was probably a factor in the sport of athletics, where for many years, a 4-minute barrier saw athletes failing to run a mile in less than 4 minutes. For years, many experts said that the human body was simply not capable of a 4-minute mile. Fatalism declared it was impossible. In the 1940’s, the mile record was pushed to 4:01, where it stood for nine years, as runners struggled with the idea that perhaps the experts had it right. Perhaps the human body had reached its limit. But on May 6, 1954, Roger Bannister broke the 4-minute barrier, running the distance in 3:59.4. Barely a year after Bannister’s accomplishment, someone else ran a mile in under 4 minutes. Then some more runners did. It has since been broken by many male athletes, and is now the standard of all male professional middle distance runners. In the last 50 years the mile record has been lowered by almost 17 seconds.

And as will be seen, fatalism can even hinder or obstruct the fight against malaria, but fortunately events sometimes present themselves which enable that fight to succeed. The Balfour Declaration had been issued by the British Government in 1917 in support of a Jewish homeland in Palestine even though the British would have been aware Palestine was drenched in malaria, and that Palestine was either uninhabitable in many areas or otherwise generally thinly populated. The only experience at that date of dealing with malaria control anywhere in the world had been demonstrated by General Gorgas at the Panama Canal together with his employment of thousands of men at vast expense, thus making it a method too costly to adopt for most countries. The severity of the disease in Palestine may be appreciated by the fact that in the final year of WWI, General Allenby’s British army was to collapse from malaria in 1918 but, fortunately for Allenby, only after first having decisively beaten the Turkish army in Palestine a few days before, in one of the final battles of WWI.

This article examines the change brought about in the then prevailing attitude to combatting malaria, which existed immediately after WWI, in 1918. Little had been done to defeat the disease in Palestine because of the view that it would be too costly, that it could only be accomplished if carried out on a large scale and that under the economic conditions then in Palestine, this was out of the question. But a Dr. I. Kligler was to introduce a change in dealing with malaria. It was a successful national malaria elimination method that was both affordable and sustainable because it relied upon the willing involvement of the Palestine population. Palestine was thereby rendered habitable.

Fatalism has been in evidence in relation to malaria elimination since the building of the Panama Canal at the beginning of the 20^th^ century, if not before. In 2017 a paper about the successful malaria elimination that began in Palestine in 1922 under the direction of Kligler was published and noted the similarity of his approach to that employed by Dr. F. Dunkel in the early 2000s in a village in Mali [1]. The paper further examined the malaria control in Palestine under General Allenby in 1918 during WWI, and was contrasted with Kligler’s malaria elimination that began in 1921/1922.

## 2 Malaria in pre-WWI Palestine

It is essential at this stage to appreciate the severity of malaria in Palestine 100 years ago, and the following extract from the previously mentioned 2017 paper may assist for this purpose [1]:

*Before World War I, for several centuries, Palestine had been a part of the Ottoman Empire. Palestine was so severely saturated in malaria, it was either uninhabitable in many areas or otherwise very thinly populated. The disease had decimated the population to the point that Mark Twain in 1867 wrote on his visit to Palestine, “A desolation is here that not even imagination can grace with the pomp of life and action…We never saw a human being on the whole route”.*

*In its 1876 Handbook for Palestine and Syria, the travel agent Thomas Cook and Son said of Palestine that “Above all other countries in the world, it is now a land of ruins. In Judea it is hardly an exaggeration to say that…for miles and miles there is no appearance of present life or habitation, except the occasional goatherd on the hillside, or gathering of women at the wells, there is hardly a hill-top of the many within sight which is not covered with the vestiges of some fortress or city of former ages”.*

*In 1902, in his report entitled “The Geographical Distribution of Anopheles and Malarial Fever in Upper Palestine,” J. Cropper wrote of Rosh Hanikra (which marked the border between the provinces of Syria and Palestine), “It was guarded by a small company of Turkish soldiers, and the platoon had to be changed every month because malaria sickened and debilitated everyone after 10 days”.*

*Between 1882–1914, approximately 75,000 Eastern European Jewish idealists arrived to settle in Palestine (not to be confused with the religious Jews who for centuries came to try to live [and die] in the Holy Land). However, by 1914, about half this number of idealist Jews had died or had left, unable to cope with the severe pestilential conditions.*

## 3 Balfour Declaration and Louis B. Brandeis

Modern Political Zionism, the movement for Jewish self-determination, arose in the late 19^th^ century as a reaction to anti-semitic and exclusionary nationalist movements in Europe. The 1881-1884 anti-Jewish pogroms in Russia stimulated the growth of Zionism, resulting in the formation of pioneering organisations and the first major wave of Jewish immigration to Palestine. In 1897, the Zionist Organisation was founded, and, at its first congress, called for the establishment for a home for the Jewish people in Palestine. During WWI, on 2^nd^ November 1917, the British Government issued a public statement, the Balfour Declaration, announcing support for the establishment of a ‘national home for the Jewish people’ in Palestine. The declaration was contained in a letter dated 2 November 1917 from the United Kingdom's Foreign Secretary Arthur Balfour to Lord Rothschild, a leader of the British Jewish Community for transmission to the Zionist Federation of Great Britain and Ireland.

Relatively late in life, an American lawyer, Louis B. Brandeis, had become a prominent figure in the Zionist movement, becoming active in 1912 in the Federation of American Zionists. He was subsequently, in 1916, to become a member of the Supreme Court, and his involvement provided the nascent American Zionist movement one of the most distinguished men in American life and a friend of the next American president. Over the next several years, he devoted a great deal of his time, energy, and money to championing the cause. A Provisional Executive Committee for Zionist Affairs was established in New York on August 20, 1914, and Brandeis was elected president of the organization. As president from 1914 to 1918, Brandeis became the leader and spokesperson of American Zionism. He worked to garner support for the Zionist cause, emphasizing the goal of self-determination and freedom for Jews through the development of a Jewish homeland. Brandeis was later to recall the effect on him of the Balfour Declaration when he spoke to an informal conference of the New England members of the Palestine Development League in 1923:

*‘Ten years ago the Homeland was a dream – a dream for which realization seemed so far. Then, we could do little more than hope and prepare ourselves for realization. Five years ago [in 1918], with [the previous year’s] Balfour Declaration, that dream began to take on the shape of opportunity. Now, for over four years the opportunity has been ours.’* [2].

Jacob De Haas, a close working-colleague and biographer of Brandeis, wrote:

*‘Brandeis made the issuance of the Balfour Declaration an opportunity for immediate action …. No publicity was given … at the beginning of 1918 [of] a group devoted to the careful study of the resources of Palestine, and a survey from historic sources of the boundaries of Palestine together with an estimate of the boundaries that in view of economic conditions would best serve the purpose of a large Jewish settlement.’* [2].

This study and survey was however noted by the Palestine Exploration Fund in London in its October 1918 Quarterly Statement which wrote:

*‘An interesting activity has been inaugurated by the Zionist Organisation of America, namely, the compiling of a bibliography of literature on Palestine, consisting of a catalogue raisonne of books, articles, pamphlets, etc., on Palestine in all languages. Special stress is being laid on economic rather than archaeological material. For further information address, ‘Palestinian Survey, 500, fifth Avenue, New York, USA.’* [3]

It may assist to illustrate the nature and extent of this study and examination of Palestine by listing the topics covered in these studies and surveys for the Zionist Organisation of America, and for which the following individual reports [4] by different experts were prepared:

- The Boundaries of Palestine
- A Sketch of the Geography of the Holy Land
- A Rapid Sketch of the History of Archeological Exploration in the Holy Land
- Preliminary Report on the Meteorology of Palestine
- Preliminary Report on the Geology of Palestine
- Soil Problems in Palestine
- Oil as Fuel in Palestine
- The Control of Plant and Animal Pests
- Sanitary Survey of Palestine [5]
- Memorandum for the Palestinian Survey on the Fiscal System and Financial Distribution in Palestine
- Home Industries in Palestine
- International Zion
- A Survey of Palestinian Libraries

Armed with these Reports and with the close of WWI, Brandeis visited Palestine in 1919, and he was greatly impressed by the seriousness of the malaria situation. Brandeis suffered with malaria, and his boyhood experience with malaria in Kentucky (where he had contracted the disease) had left a deep impression on him. And this, coupled with his practical sense made him grasp the significance of the problem that faced the new settlers. He realised that before all else, the land had to be made safe for settlement. De Haas wrote [2]:

*‘But a practical question, suggested by the bloated appearance of hundreds of little children, distressed him. The land was filled with malaria, and he knew malaria and its evil influence from his Kentucky boyhood, when the bowl of quinine pills was always on the table. To put an end to malaria was therefore the first task he would assign to the American Zionists.’*

Upon his return to the United States, Brandeis proceeded directly to a Zionist convention held in Chicago. De Haas continued [2]:

*‘The delegates [to the convention] were elated by Brandeis’ first words and exalted by the spirit of his address. But they were dumbfounded when suddenly he turned from this high idealism and outlined a specific action, the stamping out of malaria in Palestine. Malaria had been regarded as so natural an incident to Palestinian life that few considered it as a serious evil. … The delegates did not perceive that Brandeis was calculating the economic loss due to malaria, …. Therefore they did not respond to Brandeis’ suggestion.*

*… The convention did however listen attentively in executive sessions to the constructive ‘message’ brought by Brandeis from Palestine. His program, based on the conclusion ‘that the period of practical preparatory work has begun,’ … [commenced with] – A campaign against malaria, to be waged vigorously in advance of any extensive immigration.*

*… The Brandeisian view was the controlling influence at [this] convention. But as the Zionists did not in practice immediately respond to this code, Brandeis made the stamping out of malaria a personal objective [to enable a fulfilment of his Zionist dream]. It is thanks to his support of the scientists who did the work, that this scourge has disappeared throughout the largest part of Palestine.’*

Reverting to the Balfour Declaration and malaria elimination, the connection made by Brandeis may be seen also in a letter he wrote on 22^nd^ September 1919 to Adolph Kraus where Brandeis wrote:

*‘Now that the future of the country, as a Jewish Homeland, seems assured [due to the Balfour Declaration] and political questions are disposed of, all members of the B’nai Ivrith should be ready to take part in preparing the land for Jewish immigration*.’ [6].

And as if to again remind of Brandeis’ condition precedent for Jewish immigration, a letter of 24^th^ September 1919 to Jack Mosseri by Brandeis stated:

*‘We in America are planning how to direct our attention to the development of Palestine and to prepare it for receiving the immigrants who are pressing for admission. To this end an anti-malaria campaign is the first step*.” [6].

Kligler, a brilliant public health scientist working for the Rockefeller Institute and an admirer of Brandeis, was the author of one of the above 1918 Brandeis Reports entitled ‘Sanitary Survey of Palestine’ [5]. In 1925, he was afterwards to write of what he knew of the experience of Brandeis in 1919 in an article entitled *‘*The Fight against Malaria’ for the Menorah Journal:

*‘Most amazing was the resignation with which these terrible conditions [in Palestine] were accepted by all engaged in the Zionist enterprise – settlers, prospective settlers and administrators alike. When, after his visit to Palestine [in 1919], Justice Brandeis tried to arouse the Zionists to the seriousness of the situation, he was attacked as a maligner of the good name of the country. But Brandeis knew at first hand the effects of malaria, for he had it himself; he had travelled over the entire country of Palestine and seen the inroads the disease was making in the vigour and productivity of the settlers**.** Yet his recommendations were condemned as visionary.*’ [7].

The acceptance of the disease was commonplace throughout the world 100 years ago and was (and still is even today) a huge obstacle to malaria elimination. Fatalism, the sense of inevitability of the disease, was a principal barrier to malaria elimination. In 1925, Kligler wrote sarcastically of the prevailing negative attitude in the early 1920s towards the malaria situation within the Zionist establishment generally and its justification for inaction:

*‘You can get rid of the malaria [in Palestine] only by extensive drainage; extensive drainage will require enormous sums (millions were mentioned), [therefore] malaria cannot be eliminated from the country. Q.E.D*.’ [7]

But Brandeis’ insistence that malaria elimination should be treated as a priority was exceptional and inspired. He was indeed a visionary.

In 1919 Brandeis broke with Chaim Weizmann, the leader of the European Zionism over a number of Zionist matters, and in 1921 Weizmann's candidates, headed by Louis Lipsky, defeated Brandeis's for political control of the Zionist Organization of America.

## 4 Kligler’s attitude before and after his arrival in Palestine

Kligler’s report for Brandeis, prepared in 1918 before he travelled to Palestine, was based on literature, articles, books etc. available in 1918, and prepared by scientists, travellers etc. to Palestine before WWI. And so it is unsurprising that Kligler wrote of the malaria position, merely expressing the then current view of the day:

*‘[A mosquito control] campaign may be directed against the adult mosquitoes in houses etc, against the larvae in the ponds and swamps. But the really effective method is to destroy the breeding places. The measures to be adopted are those so successfully employed by Gen. Gorgas in Panama and Cuba. …. The campaign as outlined will no doubt require a large outlay of money and energy. … The criterion for success in building the Panama Canal was the eradication of the mosquito; the same criterion conditions the success in rebuilding our home in Palestine*.’ [7].

In 1918, there was then no proven, affordable method that rendered severely malarial land safe and usable. The general experience in the world of malaria control involved thousands of men (as demonstrated e.g. by Gorgas at the Panama Canal) thus causing such control to be at vast cost and expense. Therefore, malaria elimination which was both sustainable and affordable was unknown in those days. By way of illustration of the expense of maintaining malaria control at the Panama Canal, a paper by Henry Kumm of the International Health Division, Rockefeller Foundation, for the 1941 Symposium in America on Human Malaria [8] pointed out:

*‘A malaria control program has been in operation in the Panama Canal Zone for the past 35 years and the number of employees has at times exceeded 50,000.*’

Unbeknown to Kligler as he had been writing his report in 1918, sustainable malaria control in Palestine was being viewed by the British governing authorities as impossible. A 1918 British Army Medical Authority report noted that:

*‘It is interesting to speculate on what can be the future of a country such as [Palestine] from the health point of view. One cannot conceive the [malaria] problem [in Palestine] which faced the Army last spring [in 1918 during WWI] being undertaken by a Civil Authority. The expense alone would be prohibitive… The great bulk of the work [carried out by the British Army] was washed out by the first rains of October (1918*)’. [9].

Allenby had identified the importance of first managing malaria in waging war in a malarious zone, and that the key focus of that management had to be destruction of the mosquito breeding sites. But in 1918 it was still thought that only money and thousands of personnel could sustain this type of malaria elimination, as no-one had yet considered, as an alternative, involvement of the population in the antimalarial works. After the defeat of the Ottoman army in 1918, the Palestine Mandate on behalf of the League of Nations was operated from 1920 to 1948 by a British civil administration.

In December 1920, Kligler went to Palestine to direct the Laboratories of the Hadassah Hospitals and also with a view of coming to grips with the malaria situation. After arriving and quickly studying the situation, he agreed with Brandeis that if malaria could not be eliminated in Palestine, a Jewish Homeland there was in all probability impossible. Kligler later wrote in the 1925 Menorah Journal [7]:

*‘Very little personal investigation of Palestine was necessary to convince me of the truth of Justice Brandeis’ contention that unless something was done to check the ravages of malaria, the reconstruction of Palestine [as was the Zionist dream] would be a costly if not altogether an impossible effort. How to approach the problem was a more difficult matter. ….. But I suspected then, and am now convinced, that even had large sums been available for drainage and the drainage accomplished, the malaria would have been little affected, because mosquitoes breed in little, out-of-the-way unsuspected places, which even the most elaborate systems of drainage will not reach. And at least half of the malaria can be ascribed simply to human carelessness and neglect.*

*It seemed best to begin with a modest experiment in malaria control in two or three highly infected sections of the country. … A detailed plan and approximate budget were sent [in 1921 to the USA] and the plan was approved after some discussion. Justice Brandeis personally contributed the money required - $10,000 immediately and another similar sum if needed. $10,000 however was more than sufficient for the purpose.*

*The experimental demonstrations in malaria control were conducted in three localities: …. No radical drainage was attempted: … The campaign was carried out along these main lines:*

1. *Detection and treatment of carriers*
2. *Anti-mosquito campaign. This was aimed principally at the larvae [and destruction of their breeding sites]*
3. *Quinine prophylaxis [hardly used]*
4. *Education. This phase was particularly stressed: by means of illustrated lectures on malaria, its causes, prevalence, and modes of prevention; by illustrated pamphlets; and by personal interviews and visits to delinquent families by local malaria inspectors. Palestine had its own Health Day with lectures, visits to breeding places and demonstrations of methods of control.*

*First, and perhaps the most important of the fruits of this modest experiment, was the change in the attitude of the population towards malaria. ….. Even in advance of the evidence of figures and charts the attitude of the population had changed; they had come to realise that malaria was a preventable disease.’*

Subject to the success of the experimental demonstrations, Kligler’s plan for malaria elimination had been to be principally focused on destruction of the breeding sites of the mosquito which carried the disease. His proposed method included engaging with the whole rural Palestine population (albeit this was very small) to eventually secure the cooperation of both Arab and Jewish local communities who would also maintain the anti-malaria works which he intended would be carried out, and thereby ensure the mosquito did not return to that district.

Kligler’s significant change in approach against the disease was to think not of malaria control and use of thousands of employed personnel, but to seek instead malaria elimination through involvement of the population by culturally-sensitive education. Without Brandeis’ personal financial contribution towards the experimental demonstrations, Kligler could never have demonstrated the success of his approach. And subsequently, as a result of the successful demonstrations, future funding was secured to begin malaria elimination coverage of the whole country.

## 5 Success of Kligler’s approach to malaria elimination

In 1924, the Malaria Commission of the League of Nations, the forerunner of the United Nations, had been unaware of the anti-malaria works in Palestine. The Commission had stated it didn’t know what to suggest with regard to malaria elimination. It had visited/inspected Serbia, Croatia, Slovenia, Greece, Bulgaria, Romania, Russia (Ukraine, Caucasus, Volga region, Moscow) and Italy (Venice, Turin, Rome) and the subsequent 1924 Commission Report stated:

*‘… in all the countries visited, there are certain areas in which endemic malaria has always existed as a more or less common disease; … In considering how the European malaria situation can best be met, we have to acknowledge… that in reality we are not in a position to suggest any single plan for dealing with malaria which would certainly be permanently effective in actual practice.’* [10].

In 1925, the League of Nations Malaria Commission heard of anti-malaria works being conducted in Palestine and visited to inspect. The Commission subsequently reported:

*‘Palestine is a small country and, as a whole, thinly populated. … malaria … has always been very prevalent, particularly at Jerusalem … at Jaffa, Acre … and in the Valley of the Jordan*’[11],

but the Commission was so impressed by what it saw that it concluded the Report of its inspection with:

*‘…the work done in Palestine destroyed pessimism, raised hopes…’* and *‘the men who carried it out can be regarded as benefactors not only to the Palestinian population but to the world as a whole.’* [11].

And despite the incitement and resulting troubles and violence, during the 1930s, in each year, the British Health Department in Palestine repeatedly praised the strong cooperation of Arabs and Jews that existed, and in 1941, the Health Department reviewed the position with the following comment:

*‘As the general scheme has gradually advanced in scope, so the community self-help which has been stressed already as a particular feature of the antimalarial scheme here has come more and more to the fore. … In rural areas, all headmen and villagers and settlers, must cooperate in the cleaning and channelling of the more important streams and other water holding places adjacent to their dwellings, under skilled government supervision. This is now a seasonal procedure after the April rains. … such co-operation was willingly, and even enthusiastically, given. For as the health of villagers and settlers improved from year to year, as dunnum after dunnum of waste land was gradually added to the use of farmers and shepherds, so did this co-operation steadily increase in volume and energy.… that no actual and serious damaging effect on the community as a whole has resulted from these troubles (disturbances) is a matter for satisfaction: a result due, without doubt, to the system of observation, and wide and detailed control of the disease, now practised in all the most populous and important sections of the country.”* [9].

Such tributes and praise could never have been paid without that initial defeat of fatalism. Such co-operation could never have survived unless the population from the outset was committed to the project and believed in it. This must have been very much in evidence for the League of Nations to have taken the trouble to comment that it ‘destroyed pessimism, raised hopes’.

## 6 Conclusions

A century ago, Palestine was drenched in malaria. But in 1922, it became the place of the first start anywhere in the world of a successful national malaria elimination campaign. The first breach of the ‘4-minute mile’ fatalism barrier in malaria-elimination began 100 years ago in Palestine. Absence of fatalism is essential for successful, sustainable malaria elimination and it is intended that this article may hopefully serve as an example of the expression ‘If you will it, it is no dream’, and thereby stimulate an interest to examine how Kligler engaged with the population.
